# Development of a novel prognostic signature for predicting the overall survival of bladder cancer patients

**DOI:** 10.1042/BSR20194432

**Published:** 2020-06-10

**Authors:** Huamei Tang, Lijuan Kan, Tong Ou, Dayang Chen, Xiaowen Dou, Wei Wu, Xiang Ji, Mengmeng Wang, Zengyan Zong, Hongmei Mo, Xiuming Zhang, Dan Xiong

**Affiliations:** Medical Laboratory of the Third Affiliated Hospital of Shenzhen University, Shenzhen 518001, China

**Keywords:** bladder cancer, overall survival, prognostic signature

## Abstract

Background: Bladder cancer is one of the most common malignancies. So far, no effective biomarker for bladder cancer prognosis has been identified. Aberrant DNA methylation is frequently observed in the bladder cancer and holds considerable promise as a biomarker for predicting the overall survival (OS) of patients. Materials and methods: We downloaded the DNA methylation and transcriptome data for bladder cancer from The Cancer Genome Atlas (TCGA), a public database, screened hypo-methylated and up-regulated genes, similarly, hyper-methylation with low expression genes, then retrieved the relevant methylation sites. Cox regression analysis was used to identify a nine-methylation site signature of a training group. Predictive ability was validated in a test group by receiver operating characteristic (ROC) analysis. Results: We identified nine bladder cancer-specific methylation sites as potential prognostic biomarkers and established a risk score system based on the methylation site signature to evaluate the OS. The performance of the signature was accurate, with area under curve was 0.73 in the training group and 0.71 in the test group. Taking clinical features into consideration, we constructed a nomogram consisting of the nine-methylation site signature and patients’ clinical variables, and found that the signature was an independent risk factor. Conclusions: Overall, the significant nine methylation sites could be novel prediction biomarkers, which could aid in treatment and also predict the overall survival likelihoods of bladder cancer patients.

## Introduction

Bladder cancer is a genomically heterogeneous malignancy with nearly 430,000 cases diagnoses worldwide and over 165,000 deaths from this disease in 2012 [1]. Urothelial carcinoma accounts for >90% of all bladder cancers and is classified into two clinical phenotypes: superficial nonmuscle-invasive bladder cancer and muscle-invasive bladder cancer [2,3].

A variety of factors, such as environmental or occupational exposure to tobacco and biological infection by Schistosoma haematobium, were proved to be associated with the onset of bladder cancer [4,5]. Some hereditary genetic alterations that are involved in carcinogen bioactivation and detoxification may possibly make an individual more susceptible to extrinsic carcinogens [6,7]. Moreover, the accumulation of genetic changes and epigenetic alterations may contribute to the initiation and progression of human cancers, including bladder cancer [8–11].

DNA methylation is conventionally negatively associated with gene expression [12]. The dynamic regulation of DNA methylation is a key process involved in a wide variety of functions that include the maintenance of genomic integrity and stability, gene regulation and cellular differentiation [13,14]. In cancer and other pathological conditions, the methylation levels of DNA may change to hypo-methylation (the state of decreased methylation) or hyper-methylation (the state of increased methylation) level [15]. Therefore, DNA methylation-based biomarkers hold considerable promise for predicting the biological behavior of cancer and the survival of patients [16].

The Cancer Genome Atlas (TCGA) is one of the most comprehensive sequencing databases of cancer, which contains methylation data for 28 cancers [17]. In the present study, transcriptome and methylation data for bladder cancer were downloaded from the TCGA. Using a series of statistical methods, we screened a nine-gene site signature that was significantly correlated with bladder cancer patients’ overall survival (OS) in a training group, established a risk score system based on the signature and illustrated the risk score in a nomogram. We also validated the signature with information from a test group. Furthermore, take the clinical characteristic into consideration, a more comprehensive nomogram was constructed that integrated the clinical characteristics.

## Materials and methods

### Data retrieval and screen differently methylated genes (DMGs)

Level 3 transcriptome data and DNA methylation profiles were downloaded from TCGA (http://cancergenome.nih.gov/) data portal using the R project (version 3.5.1, http://www.r-project.org/) [17]. DNA methylation data contained preprocessed data in the form of β values, which indicate the degree of methylation. DNA methylation data evaluated on the Illumina Infinium Human Methylation 450 platform (San Diego, CA) were included in our study. We identified differentially expressed genes (DEGs) by using the edgeR package and DMGs by using the Limma package in the R software with fold-changes ≥ 2 [18]. *P* values and false discovery rates (FDRs) less than 0.05 were considered significant [19]. We screened genes coexisting in the two groups with overlapping hypo-methylated and up-regulated genes, similarly, hyper-methylation with low expression genes, then retrieved the corresponding methylation sites of the genes.

### Functional and pathway enrichment analyses of the DMGs

Biological annotation of DMGs was performed using the Database for Annotation Visualization and Integrated Discovery (DAVID, https://david.ncifcrf.gov/), which is a widely used web-based tool for analyzing enriched Gene Ontology (GO) terms and pathways. In the present study, it was used to perform GO and Kyoto Encyclopedia of Genes and Genomes (KEGG) pathway enrichment analyses of the DMGs. Moreover, the criterion for significant enrichment of GO terms and KEGG pathways was *P* value < 0.05.

### Formulation of DMGs signatures

Bladder cancer samples from patients with clinical characteristics and survival data were randomly divided into two groups: a training group (265 patients) and a test group (133 patients). We performed a univariate Cox proportional hazards regression analysis to assess the correlation between patient OS and methylation sites in the training group with *P*<0.5. To access a more powerful predictive diagnostic and prognostic signature, we performed a multivariate Cox regression. The model with minimum AIC was screened by R. A risk scoring formula was construct based on the Cox regression analysis. The risk scoring formula is shown in the following equation: Risk Score $(\text{RS})=\sum_{i=1}^{N}\left(Exp*Coef\right)$, which was used to calculate the signature RS for a patient. *N* is the representative number of prognostic DNA methylation sites; Exp is the degree of DNA methylation; and Coef is a single factor means Cox regression coefficient. Hazard ratios (HRs) and 95% confidence intervals (CIs) were calculated based on the Cox regression analysis. Moreover, a nomogram model was established based on the results of the multivariate Cox regression analyses to predict 1-, 2- and 3-year OS. The performance of the nomogram was evaluated by Harrel’s concordance index (C-index), which comparing the predicted and actual probabilities for OS. The value of the C-index ranges from 0.5 to 1.0, the closer to 1.0 the better ability to correctly discriminate the outcome with the model [20]. To provide more comprehensive guidance for clinical diagnosis and treatment, multivariate Cox regression analysis was used to establish a nomogram that integrated the risk scoring model and the clinical information.

### Statistical analysis

Receiver operating characteristic (ROC) and Kaplan–Meier survival analyses were used to validate prognostic ability of the DNA methylated site signature in the training and test groups. We used ROC curve to analyze the specificity and sensitivity across a range of cutoff points for the predictive signature and calculated the area under curve (AUC). We chose the median risk score as a cutoff point in the training and test cohort and divided the bladder cancer patients into low- and high-risk groups. Kaplan–Meier survival analysis was used to estimate the OS of the two groups, and log-rank test was used to evaluate the statistical significance.

## Results

### Data processing and patient characteristics

**Figure 1:**
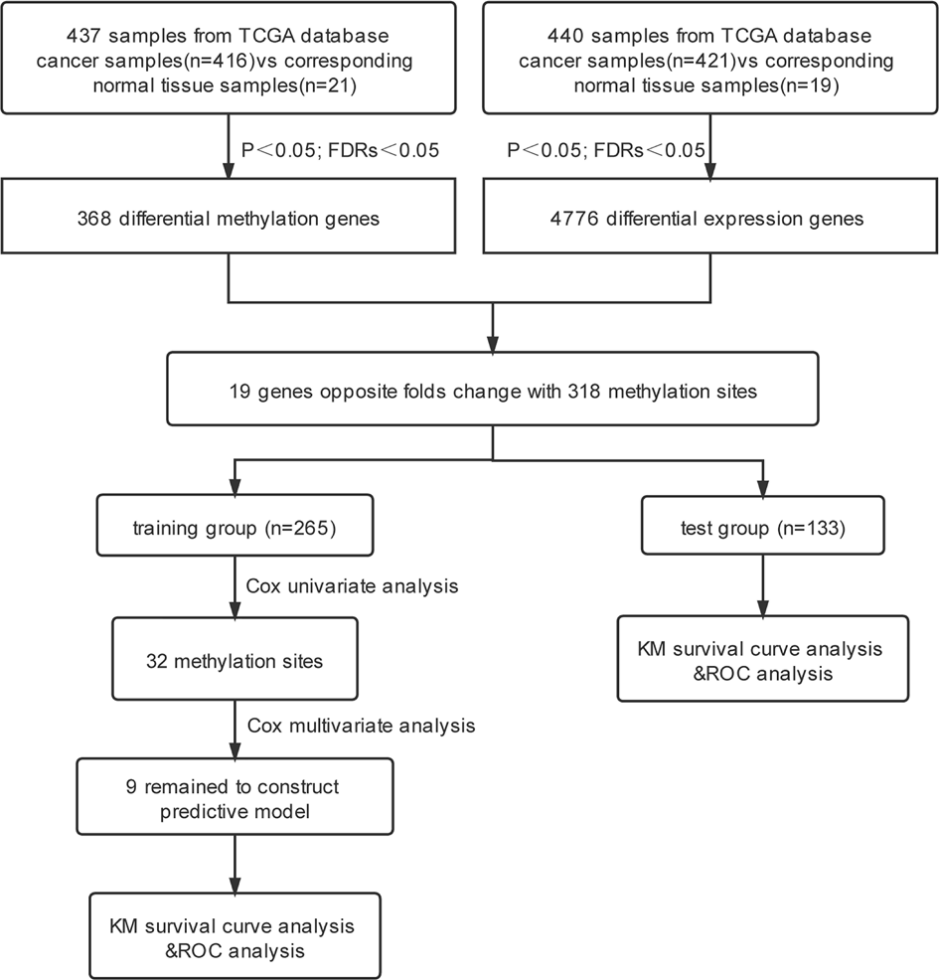
Flowchart of the research The flowchart showing the scheme of developing the prognostic gene signature and validating its efficiency to predict survival. FDRs, false discovery rates; KM, Kaplan-Meier; ROC, receiver operating characteristic; TCGA, The Cancer Genome Atlas.

The transcriptome profile included RNA sequencing data from 421 bladder cancer samples and 19 normal tissue samples; the methylation dataset contained 416 bladder cancer samples and 21 adjacent tissues. Among them, 409 samples had clinical information, such as sex, age and stage. A total of 398 patients clinically diagnosed with bladder cancer were randomly divided into a training group (*n*=265) and a test group (*n*=133). According to the International Union Against Cancer tumor-node-metastasis classification system for bladder tumors, clinical stages of the tumors were classified into stages I to IV (Table 1). The flowchart for the present study is shown in Figure 1.

**Table 1:** Summary of patient demographics and characteristics

Characteristic	Training (*N*=265, **three patients without stage information**)	Test (*N*=133, one patient without stage information)
Gender		
Female	65 (24.5%)	42 (31.6%)
Male	200 (75.5%)	91 (68.4%)
Age		
<60 years	64 (24.2%)	25 (18.8%)
≥60 years	201 (75.8%)	108 (81.2%)
Stage		
I	2 (0.8%)	2 (1.5%)
II	77 (29.1%)	46 (34.6%)
III	89 (33.6%)	45 (33.8%)
IV	94 (35.5%)	39 (29.3%)
Vital status		
Living	165 (62.3%)	75 (56.4%)
Dead	100 (37.7%)	58 (43.6%)

### Identification and enrichment analysis of DMGs

**Figure 2:**
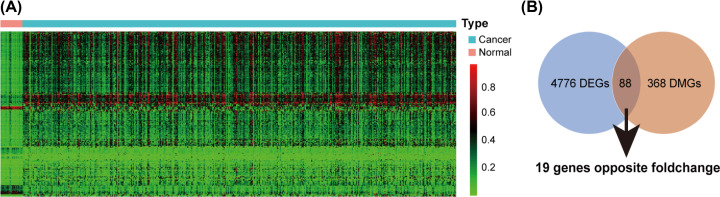
Identification of DMGs in bladder cancer (**A**) Heat map depicting methylation levels of genes in cancer and normal samples. The columns represent the cases, pink represent the cancer samples and green means the control. The lines represent the genes. Red color indicates hyper-methylation levels; green color indicates hypo-methylation level. (**B**) Venn diagram showing there were 88 genes overlapped between DEGs and DMGs, among them 19 genes with opposite folds changed; DEGs, differentially expressed genes; DMGs, differently methylated genes.

**Figure 3:**
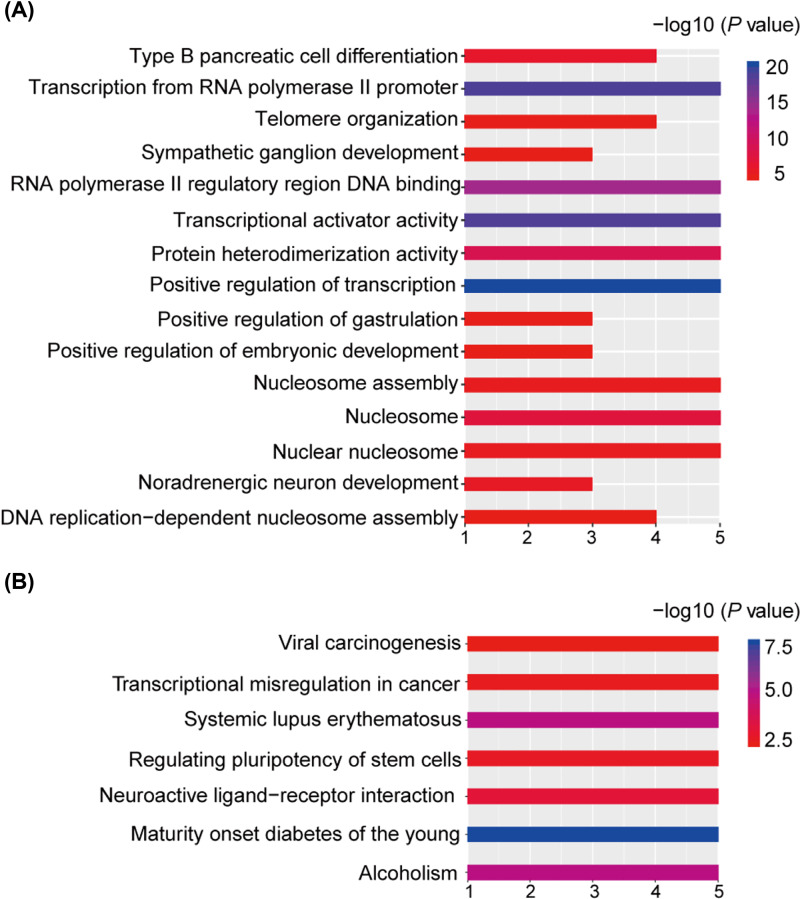
Gene Ontology (**A**) and KEGG Pathway (**B**) enrichment analysis of DMGs Horizontal axis represent the amount of genes enriched on the pathway.

We identified 4776 DEGs from the expression profile and 368 DMGs from the methylation profile, of which 88 genes were overlapped and 19 genes had inverse relationships between methylation and expression, which indicated the hypo-methylated with up-regulated genes as well as hyper-methylation with low expression genes (Figure 2). To access more information about the DMGs, enrichment analysis with the DAVID was used to elucidate their biological functions [21]. The top significant terms emerging from the GO analysis are shown in Figure 3A. DMGs were enriched in the ‘transcription from the RNA polymerase II promoter’, ‘positive regulation of transcription’ and ‘transcriptional activator activity’ terms, which suggested that DMGs may play important roles in regulating transcription in bladder cancer. KEGG analysis indicated that DMGs were significantly enriched in pathways related to cancer, such as ‘viral carcinogenesis’ and ‘transcriptional misregulation in cancer’ (Figure 3B).

### Identifying DNA methylation gene sites in the training group

**Figure 4:**
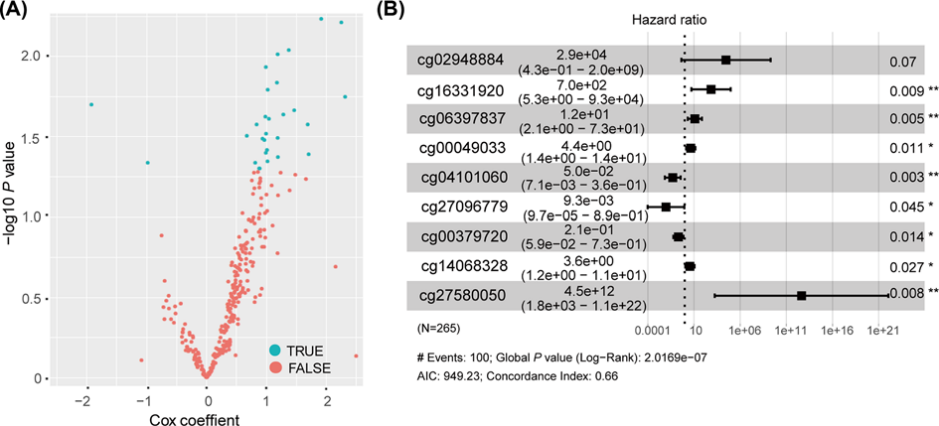
Identification of the methylation sites in the training group (**A**) Cox univariate analysis of the genes’ methylation profiling data. Genes with *P*<0.05 were screened. (**B**) Identification of nine methylation sites by Cox multivariate regression analysis. The hazard ratio and 95% confidence interval was showed next to the locus on the picture. AIC shows the fitness of model, concordance index indicates the ability of discriminating; AIC, Akaike information criterion.

About 318 methylation sites were located within the 19 selected DMGs. We screened 398 bladder cancer patients with clinical information, such as the sex, age and clinical stage of patients, and randomly divided them into a training group (*n*=265) and a test group (*n*=133). The training group was used to explore the relationship between OS and methylated gene sites. A Cox univariate analysis of the methylation sites with OS was performed, and 32 methylation sites were showed a significant correlation with OS (*P*<0.05, Figure 4A). We next used a Cox multivariate regression analysis to access an optimal prognostic model, composed of cg02948884, cg16331920, cg06397837, cg00049033, cg04101060, cg27096779, cg00379720, cg14068328, cg27580050 from the 32 methylation sites with the minimum Akaike information criterion (AIC) and *P*<0.05 (log-rank test; Figure 4B). Harrel’s concordance index (C-index) for OS prediction was 0.66. In general, a predictive model with a lower AIC shows a better model fit, while a higher C-index indicates a better discriminating ability. The nine methylation sites were closely related to patient survival. Six DNA methylation sites were presumed to be risk factors (HR > 1), while three were protective factors (HR < 1) (Figure 4B). A risk score formula was constructed to assess the total risk as follows:

Risk score = (10.28 * methylation level value of cg02948884) + (6.56 * methylation level value of cg16331920) + (2.52 * methylation level value of cg06397837) + (1.49 * methylation level value of cg00049033) + (−2.99 * methylation level value of cg04101060) + (−4.67 * methylation level value of cg27096779) + (−1.58 * methylation level value of cg00379720) + (1.28 * methylation level value of cg14068328) + (29.13 * methylation level value of cg27580050).

### Prognostic value of the nine-methylation site signature in the training and test groups

**Figure 5:**
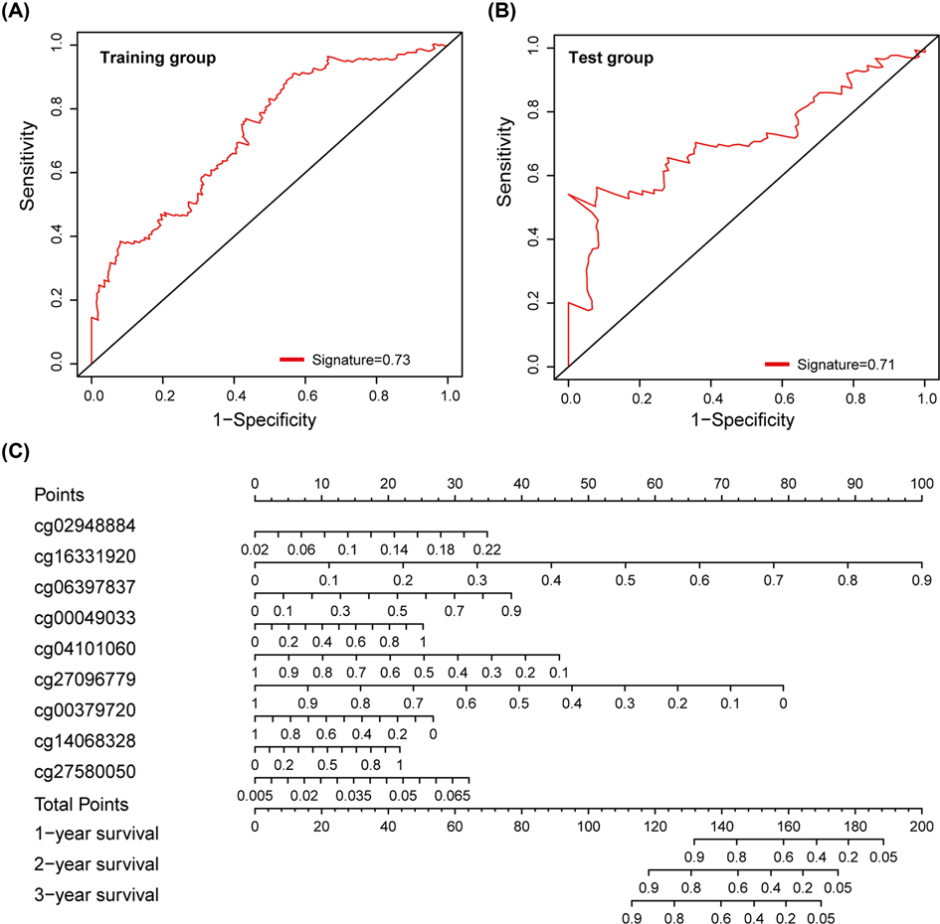
Predictive ability of the DNA methylation site signature and establishment of a nomogram for bladder cancer (**A**) ROC analysis of training group, the AUC was 0.73. (**B**) ROC analysis of training group, the AUC was 0.71. (**C**) Nomogram including nine methylation sites for predicting OS of bladder. Point of every methylated site was got according to the line drawn upward. The total points of the nine components of an individual patient calculated on the ‘Total Points’ downward, which corresponds to the probability of 1-, 2-, 3-year survival plotted below; AUC, area under curve; OS, overall survival; ROC, receiver operating characteristic.

To validate the predictive power of the methylation site signature, we conducted a separate ROC analysis in the training and test groups, considering that the larger AUC of ROC calculated a better model for prediction. Figure 5A,B shows the ROC analysis of the training group and the test group, respectively, with AUC values of 0.73 and 0.71, which demonstrated that the predictive ability of the nine-methylation site signature was high and further indicated that the signature in our study was a novel and highly accurate prognostic model.

### Establishment of a nomogram for OS prediction in bladder cancer

We developed a bladder cancer nomogram, accepted as a reliable tool to construct a simple intuitive graph of a statistical predictive model that quantified the risk of every patient, to predict a specific individual’s probability of 1-, 2- and 3-year survival based on the nine-methylation site gene signature. For every site, the value was evaluated on the scale equal to Exp * Coef. Exp is the degree of DNA methylation, and Coef is the corresponding Cox regression coefficient. The variable got the point corresponding to the point axis upward. We estimated median 1-, 2-, and 3-year survival rates by the total points downward (Figure 5C).

### Confirm the predictive power of the signature and construct a clinically associated nomogram

**Figure 6:**
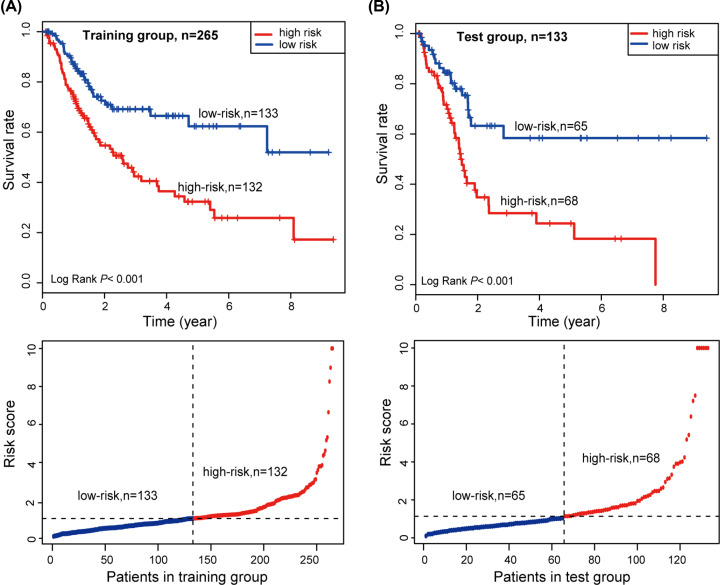
Kaplan–Meier survival curve analysis and risk score of the patients (**A**) According to the median risk score, the training group was classified into high- and low-risk groups. Kaplan–Meier survival curve analysis shows the survival rates of the high- and low-risk groups. *P* values was calculated by log-rank test. (**B**) According to the median risk score, the test group was classified into high- and low-risk groups. Kaplan–Meier survival curve analysis shows the survival rates of the high- and low-risk groups. *P* values was calculated by log-rank test.

According to the risk-score formula, the risk scores of all patients in the training group were calculated. We ranked the patients based on the risk scores and classified the training group patients into either a high-risk group or low-risk group using the median risk score as a cut-off point (Figure 6A, below). Through Kaplan–Meier survival analysis, we found that the high-risk group of patients had a lower median overall survival than those in the low-risk group (log-rank test, *P*<0.001, Figure 6A, upper).

To confirm the predictive ability, we applied the risk score formula to the testing group and used the median risk score as the cut-off point (Figure 6B, below). The results in the testing set were similar to those in the training set. The Kaplan–Meier survival analysis showed that the high-risk score patients had a lower median overall survival than those with a low-risk score (log-rank test, *P*<0.001, Figure 6B, upper).

**Figure 7:**
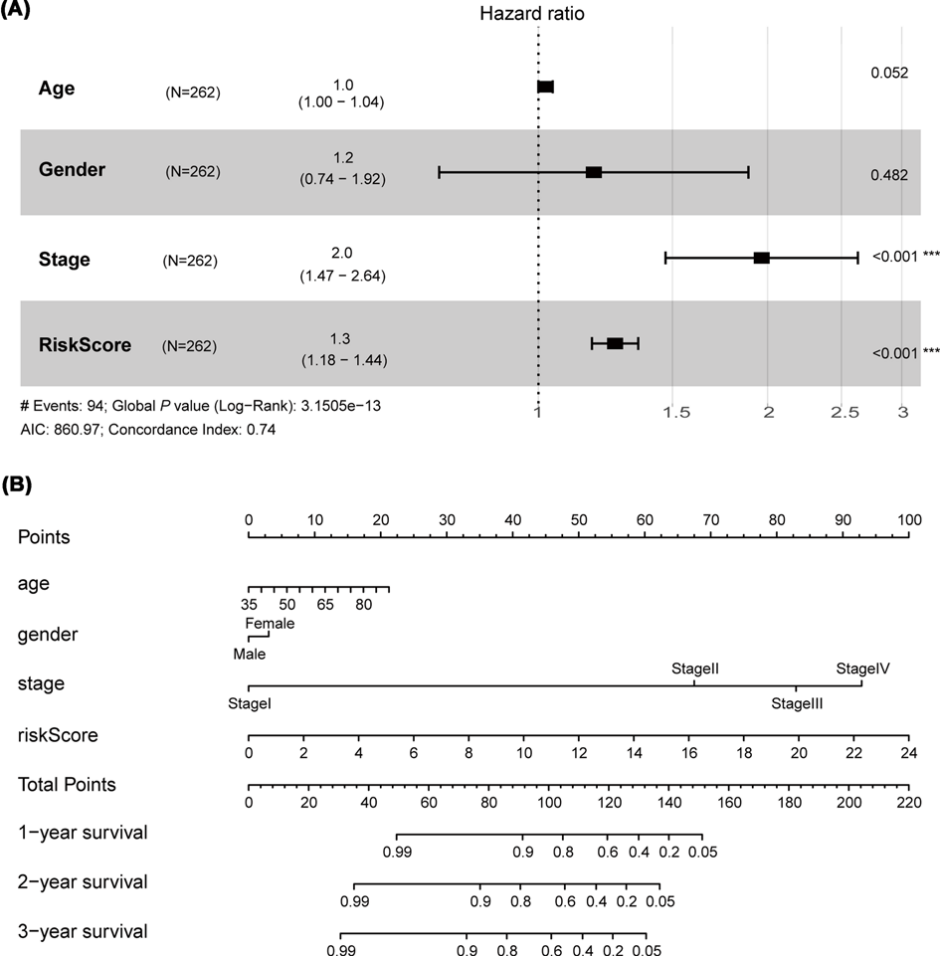
Construct a clinically associated nomogram combining clinical data and the signature (**A**) Cox multivariate regression analysis of the risk score and clinical features with OS of bladder cancer patients. The risk score integrated three clinical features, age, sex and stage were screened and construct the predictive model. (**B**) A nomogram was established based on the four factors; OS, overall survival.

To provide a clinically associated nomogram, a comprehensive nomogram was established in which the risk score integrated with three clinical features, age, sex and stage (Figure 7A). C-index of the model was 0.74, which reveal that the performance of nomogram integrated with clinical features has more powerful predictive ability than the model only based on the risk score (C-index was 0.66). We constructed the nomogram with point scales of these four variables (Figure 7B). The nomogram illustrated that the risk score sharing the largest contribution to prognosis, followed by stage. Each patient for whom with complete clinical information would obtain total points reflecting the probability of 1-, 2- and 3-year survival.

## Discussion

Numerous studies have found that epigenetic regulation, mainly aberrant DNA methylation, plays a key role in the initiation, development and progression of all human neoplasias, including bladder cancer [22]. Aberrant DNA methylation usually occurs at a GpC (a dinucleotide site that cytosine followed by guanine) island. DNA hypermethylation promotes the development or progression of bladder cancer by silencing genes involved in tumor suppression, DNA repair, cell control, cell invasion and other regulatory pathways, which occur in approximately 50–90% of bladder cancers [22,23].

In the present study, data downloaded from TCGA were used to explore the methylation of bladder cancer and 368 DMGs were identified. Enrichment analysis of the DMGs revealed they were involved in vital biological processes, including transcription from RNA polymerase II promoter, positive regulation of transcription, and transcriptional activator activity, which suggests that active transcriptions meet the needs for the rapid division of tumor cells. In addition, KEGG enrichments showed that significantly enriched pathways include viral carcinogenesis, transcriptional misregulation in cancer. Abundant expressions of BK polyomavirus genes and virus DNA integration into the host genome were detected in some bladder tumor cases, which suggested that viral infection was a critical factor in the pathogenesis, progression and metastasis of bladder cancer [24,25].

We identified nine methylation sites, which were mapped to seven genes *PITX1* (cg02948884, cg04101060), *MEOX2* (cg16331920, cg27096779), *HOTTIP* (cg06397837), *CBLN1* (cg00049033), *ZNF728* (cg00379720), *TBX18* (cg14068328) and *HSPB6* (cg27580050) by using a series of different statistical approaches. Most of the methylation sites are localized to dinucleotide CpG islands. Among the sites, both cg02948884 and cg04101060 are in the GpC island of *PITX1* (paired-like homeodomain), a tumor-suppressor gene. *PITX1* suppresses tumorigenicity by inhibiting the RAS pathway via transcriptional regulation of RASAL1, a RAS-GTPase-activating protein [26]. Liu *et al*. identified *RASAL1* as a major tumor suppressor gene in thyroid cancer with frequently hypermethylated, which was coupled with its silencing in thyroid cancer cells [27]. However, the relationship between *PITX1* and *RASAL1* in bladder cancer has remained elusive. Several studies previously found that DNA hypermethylation of *PITX1* is associated with the growth and progression of various cancers and may predict a poor prognosis [28–30]. ChIP-seq and transcriptomic analyses reveal *PITX1* as a regulator of squamous carcinogenesis in mice and humans, and *PITX1* expression controls bi-stable transcriptional circuits to govern self-renewal and differentiation in squamous cell carcinoma [31]. The transcription factor *MEOX2* (mesenchyme homeobox 2) may play a role in the regulation of vertebrate limb myogenesis. Previous studies revealed that its high expression is relevant to malignant progression and clinical prognosis in lung cancer, hepatocarcinoma and glioma, but little is known about its role in urologic tumor [32]. The Meox2/Tcf15 heterodimers modulate FA transfer to the heart and remedy cardiac dysfunction resulting from altered energy substrate usage [33]. *HOTTIP* is mainly expressed in the colon, prostate, pancreas and urinary bladder and produces a long RNA that regulates the expression of *HOXA* genes in trans. *HOTTIP* aberration promotes hematopoietic stem cell self-renewal leading to acute myeloid leukemia-like disease in mice with reprogramming leukemic-associated chromatin and gene transcription [34]. *HOTTIP* was overexpressed in small cell lung cancer (SCLC) tissues, and its expression was associated with the disease progression and the shorter survival time of SCLC patients [35]. Compared with patients with lower expression, pancreatic cancer patients with higher *HOTTIP* expression had shorter disease-free survival and overall survival [36]. *CBLN1* encodes a cerebellum-specific protein related to the tumor necrosis factor families of proteins [37,38]. *TBX18* encodes a member of the vertebrate-specific Tbx1 subfamily, which plays a crucial role in embryonic development. Combined with Tbx18, Six1 synergistically regulates smooth muscle cell development and ureter function. It is possible that Tbx18 modulates a signaling pathway that negatively regulates ureteric branching morphogenesis [39]. However, little is known about the function of *CBLN1* and *TBX18* in tumorigenesis and the progression of tumor. *HSPB6*, a small heat shock protein, plays a crucial role in muscle function and has emerged as a novel cardioprotector against stress-induced injury [40,41]. Genome-wide screening of promoter methylation has identified that the frequency of *HSPB6* promoter methylation increases moderately in early stage melanomas and significantly in advanced-stage melanomas [42]. However, not much is known about the function of *ZNF728* (zinc finger protein 728) now. Taken together, there is relatively little research on the seven signature genes in bladder cancer, and our study provides insight for further investigation into the function of these seven genes.

However, there are some limitations to our study that should be noted. Most importantly, there is a lack of information on the mechanism behind the prognostic values of these gene methylation sites in bladder cancer, and experimental studies will further enhance our understanding of their functional roles. Furthermore, although we believe that the nine-site gene signature is promising for predicting patient survival, its potential value still needs to be verified in larger groups of bladder cancer patients.

## Conclusions

Overall, our study systematically screened a DNA methylation signature for predicting of the OS of bladder cancer patients. Integrated with clinical information, we constructed a more comprehensive nomogram.

## Abbreviations

AIC: Akaike information criterion
AUC: area under curve
CI: confidence interval
DAVID: Database for Annotation Visualization and Integrated Discovery
DEG: differentially expressed gene
DMG: differently methylated gene
FDR: false discovery rate
GO: Gene Ontology
HR: hazard ratio
KEGG: Kyoto Encyclopedia of Genes and Genomes
OS: overall survival
ROC: receiver operating characteristic
TCGA: The Cancer Genome Atlas
